# Lipid Challenge Reveals Amplified Apolipoprotein Responses with Preserved Temporal Pattern in Polyendocrine Metabolic Ovarian Syndrome: An Exploratory Study

**DOI:** 10.3390/ijms27156656

**Published:** 2026-07-25

**Authors:** Ebrahim Rajab, Abu Saleh Md Moin, Manjula Nandakumar, Thozhukat Sathyapalan, Alexandra E. Butler, Stephen L. Atkin

**Affiliations:** 1 Royal College of Surgeons of Ireland, Medical University of Bahrain, Busaiteen 228, Bahrain; amoin@rcsi-mub.com (A.S.M.M.); mnandakumar@rcsi-mub.com (M.N.); abutler@rcsi-mub.com (A.E.B.); satkin@rcsi-mub.com (S.L.A.); 2 Academic Endocrinology, Diabetes and Metabolism, Hull York Medical School, Hull HU6 7RX, UK; thozhukat.sathyapalan@hyms.ac.uk

**Keywords:** polyendocrine metabolic ovarian syndrome, apolipoproteins, lipid metabolism, insulin resistance, proteomics, metabolic flexibility

## Abstract

Acute elevation in circulating lipids induces reversible insulin resistance and alters triglyceride-rich lipoprotein flux. Whether insulin-regulated remodelling of high-density lipoprotein (HDL)-associated apolipoproteins is altered during defined lipid stress in women with polyendocrine metabolic ovarian syndrome (PMOS) remains unclear. In this exploratory, randomized, cross-over study, 10 healthy women and 12 women with PMOS underwent 5-h saline or lipid infusion, with a hyperinsulinemic–euglycemic clamp commencing at 180 min. Plasma proteomics were performed at baseline, 180 min and 300 min using linear modelling (limma). Lipid infusion induced the coordinated suppression of ApoA1 and ApoE isoforms at 180 min in both groups (FDR-adjusted *p* < 0.01), followed by partial recovery during hyperinsulinemia. Women with PMOS exhibited greater early suppression of ApoA1 (log_2_ fold-change −2.20, *p* = 0.001) and more persistent suppression of ApoE isoforms at 300 min (ApoE3 log_2_ −1.64, *p* = 0.001). ApoA1 suppression correlated inversely with NEFA exposure during lipid infusion (Spearman ρ = −0.54, *p* = 0.021). Controlled lipid challenge reveals amplified but reversible dysregulation of HDL-associated apolipoproteins in PMOS, with the preservation of the overall response pattern, but impaired recovery under hyperinsulinemic conditions. These findings suggest impaired adaptation to acute lipid stress in PMOS, with ApoA1 changes appearing to relate more closely to acute NEFA exposure than to clamp-derived insulin sensitivity.

## 1. Introduction

### 1.1. Lipid-Induced Insulin Resistance as a Model of Dynamic Metabolic Stress

Metabolic flexibility, the capacity to adapt substrate utilization in response to changing nutrient availability, is a central feature of metabolic health [1]. Impairments in this adaptive response are characteristic of insulin-resistant states and contribute to the development of cardiometabolic disease [2]. Acute elevations in circulating lipids, particularly non-esterified fatty acids (NEFA), represent a physiologically relevant metabolic stress that challenges lipid handling, insulin sensitivity and inflammatory pathways [3,4].

While fasting biomarkers provide a static snapshot of metabolic status, they may not capture dynamic impairments in lipid regulation that become apparent under metabolic challenge. Controlled lipid infusion models, particularly when combined with a hyperinsulinemic–euglycemic clamp, provide a robust approach for investigating these dynamic responses in humans [5,6].

### 1.2. Lipoprotein Metabolism and Apolipoprotein Regulation

Apolipoproteins play a central role in lipid transport, lipoprotein remodelling and inflammatory signalling. Apolipoprotein A-1 (ApoA1), the principal protein component of high-density lipoprotein (HDL) that stabilizes HDL particles, is critical for reverse cholesterol transport and exhibits sensitivity to insulin-resistant states [7]. Apolipoprotein E (ApoE) is also involved in lipid trafficking and immune regulation, with expression and function modulated by metabolic conditions [8].

Together, ApoE and ApoA1 form an integrated lipid-transport network that is dynamically regulated in response to metabolic challenges [9]. Disruption of this network has been implicated in various cardiometabolic and inflammatory conditions, reflecting its central role in maintaining lipid homeostasis [9,10,11].

Although these proteins have been extensively studied in the context of neurodegenerative disease, their primary physiological functions are closely linked to systemic lipid metabolism and inflammatory regulation [11]. These observations support investigation of ApoA1 and ApoE as markers of metabolic stress beyond their original disease context. Integration of plasma proteomics with the lipid infusion-clamp model enables assessment of coordinated molecular responses to lipid-induced insulin resistance in vivo [12].

### 1.3. Metabolic Dysfunction and Lipid Stress Responses

Metabolic dysfunction, particularly insulin resistance and type 2 diabetes, is associated with altered lipid handling, increased circulating NEFA, and impaired suppression of lipolysis during insulin stimulation [4]. These abnormalities result in prolonged exposure of peripheral tissues to circulating lipids and may amplify the molecular response to acute lipid stress.

Despite growing recognition that chronic dyslipidemia contributes to disease risk [11], the effects of acute lipid elevations on circulating apolipoproteins and related proteins are not completely understood [13]. Understanding these dynamic responses may provide insight into early disturbances in lipid handling that are not evident under fasting conditions.

### 1.4. Sex Specific Metabolism and PMOS

Women exhibit sex-specific regulation of lipid metabolism, including greater reliance on lipid oxidation and distinct NEFA kinetics during insulin stimulation [14]. These features are disrupted in polyendocrine metabolic ovarian syndrome (PMOS), previously known as polycystic ovary syndrome (PCOS) [15]. PMOS affects up to 20% of premenopausal women and is characterized by insulin resistance, impaired metabolic flexibility, altered lipid handling and dyslipidemia [16,17]. Previous studies have investigated ApoA1 and ApoE in women with PMOS under fasting conditions [18,19]. However, little is known about the dynamic responses to acute lipid challenge.

### 1.5. Study Aims

While previous studies have focused on chronic metabolic abnormalities, few have examined how acute lipid exposure dynamically alters proteins involved in lipid transport and metabolic regulation [13]. To address this gap, we conducted a targeted, hypothesis-driven proteomic analysis of lipid- and insulin-responsive plasma proteins in women with PMOS and healthy controls undergoing a controlled lipid infusion combined with hyperinsulinemic–euglycemic clamp methodology [6]. The focus was on ApoA1 and ApoE dynamics as key regulators of lipid transport and lipoprotein metabolism.

The primary objective was to determine whether acute lipid exposure induces differential changes in circulating apolipoproteins and related proteins in women with PMOS compared with healthy controls. We hypothesized that women with PMOS would exhibit exaggerated proteomic responses to lipid challenge, consistent with impaired metabolic flexibility. A second objective was to assess the reversibility of these responses during insulin stimulation, as an indicator of metabolic resilience.

By characterizing the magnitude and reversibility of proteomic responses, this study aims to identify features consistent with metabolic resilience or vulnerability and to evaluate whether dynamic protein responses provide insight into lipid handling beyond static metabolic measures.

## 2. Results

### 2.1. Participant Characteristics

Ten healthy women and twelve women with PMOS completed the study and were included in the analysis. Baseline characteristics and clinical characteristics are summarized in Table 1. The groups were comparable in age and body mass index; however, women with PMOS had higher waist-to-hip ratio, elevated HOMA-IR, and lower high-density lipoprotein cholesterol (HDL-C). Fasting triglycerides and NEFA were not different between the groups (Table A2). As we previously reported, lipid infusion markedly increased plasma NEFA and triglycerides, and reduced insulin-stimulated glucose disposal in both groups, confirming the effectiveness of the metabolic challenge [20].

### 2.2. Proteomic Responses

**Figure 1:**
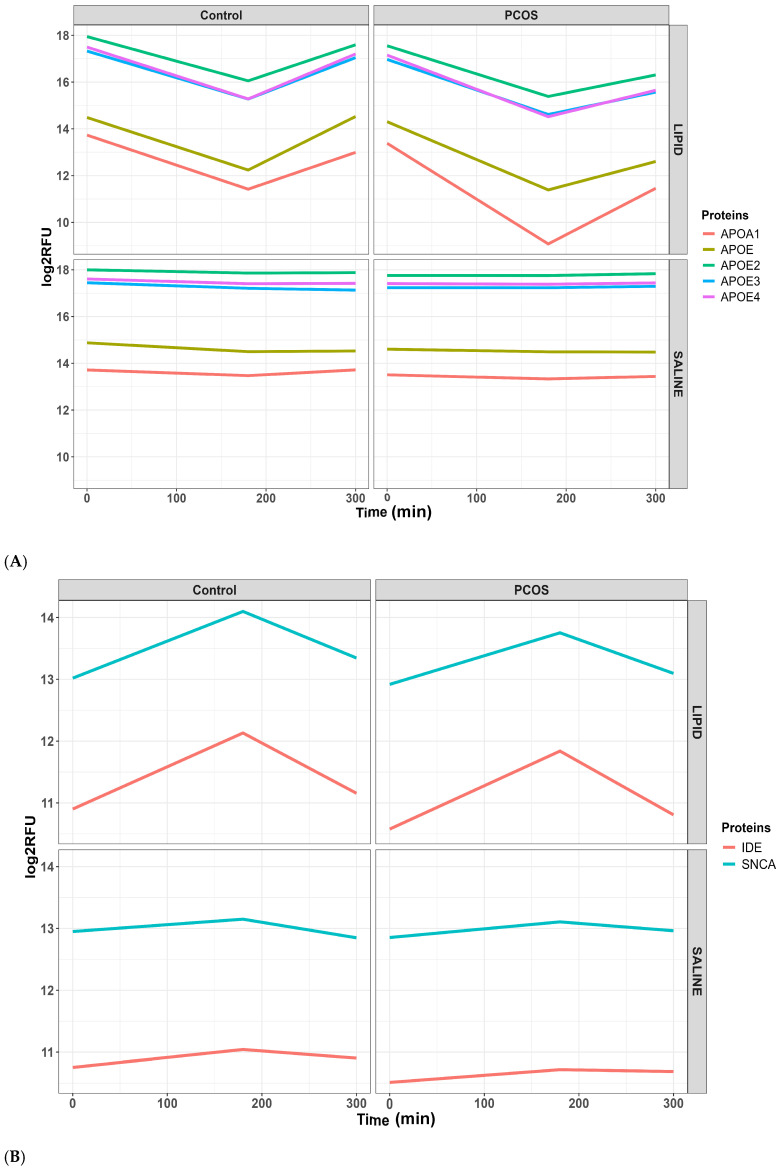
Temporal changes in selected lipid-responsive plasma proteins during saline and lipid infusion in healthy control women and women with polyendocrine metabolic ovarian syndrome (PMOS). (**A**): Apolipoprotein A1 (ApoA1), Apolipoprotein E (ApoE) and its isoforms (ApoE2, ApoE3, ApoE4). (**B**): Insulin-degrading enzyme (IDE) and α-synuclein (SNCA). Log2-transformed relative fluorescence units (RFU) are shown at baseline, 180 min (prior to insulin infusion), and 300 min (end of hyperinsulinemic–euglycemic clamp). Data are presented separately for saline and lipid infusion conditions. The figure illustrates exploratory temporal patterns of protein responses before formal linear modelling analyses presented in Table 2 and Table 3 and Table A3, Table A4, Table A5, Table A6, Table A7 and Table A8 in Appendix B.

Protein expression remained stable during saline infusion in both groups, confirming that the observed changes were lipid-specific (Figure 1).

**Table 2:** Differential response of targeted plasma proteins to lipid infusion in women with PMOS compared with healthy controls (saline-adjusted, 180 min).

Samples	logFC	AveExpr	t	Adj. *p*-Value (FDR)
ApoA1	−2.201	12.748	−3.367	0.001
ApoE4	−0.737	16.817	−1.495	0.138
ApoE3	−0.684	16.684	−1.388	0.168
ApoE	−0.843	13.906	−1.378	0.171
ApoE2	−0.561	17.313	−1.304	0.195
SAA1	−1.401	9.392	−1.165	0.247
APCS	−0.276	14.947	−1.034	0.304
PAPPA	−0.348	13.284	−1.022	0.309
APP	0.534	15.055	1.018	0.311
SNCA	−0.303	13.170	−0.855	0.394
NGF	−0.241	9.188	−0.373	0.710
GFAP	0.061	8.659	0.288	0.774
NOG	−0.135	11.741	−0.237	0.813
MAPT	0.030	6.909	0.091	0.927
IDE	0.031	10.992	0.079	0.938

Differential protein responses were estimated using linear modelling (limma), comparing lipid infusion relative to saline infusion at 180 min. Values represent log2 fold-change (FC) differences between women with polyendocrine metabolic ovarian syndrome (PMOS) and healthy controls, adjusted for within-subject saline responses. *p*-values were corrected for multiple testing using the Benjamini–Hochberg false discovery rate (FDR). Protein abbreviations: APCS, Serum amyloid P-component; ApoA1, apolipoprotein A-I; ApoE, apolipoprotein E; APP, Amyloid beta A4 protein (amyloid precursor protein); GFAP: Glial fibrillary acidic protein; IDE, insulin-degrading enzyme; MAPT, Microtubule-associated protein tau; NGF, Beta-nerve growth factor; NOG, Noggin; PAPPA, Pappalysin-1; SAA1, Serum amyloid A-1 protein; SNCA, alpha-synuclein.

**Table 3:** Differential response of targeted plasma proteins to lipid infusion in women with PMOS compared with healthy controls (saline-adjusted, 300 min).

Samples	logFC	AveExpr	t	Adj. *p*-Value (FDR)
ApoE3	−1.636	16.684	−3.322	0.001
ApoE4	−1.572	16.817	−3.187	0.002
ApoE	−1.881	13.906	−3.076	0.003
ApoE2	−1.243	17.313	−2.889	0.005
ApoA1	−1.258	12.748	−1.925	0.057
SAA1	−1.600	9.392	−1.331	0.186
APP	0.653	15.055	1.243	0.217
SNCA	−0.363	13.170	−1.025	0.308
PAPPA	−0.309	13.284	−0.908	0.366
APCS	−0.162	14.947	−0.606	0.546
MAPT	0.131	6.909	0.394	0.695
IDE	−0.135	10.992	−0.345	0.731
GFAP	−0.038	8.659	−0.180	0.857
NGF	−0.079	9.188	−0.122	0.903
NOG	0.003	11.741	0.005	0.996

Differential protein responses were estimated using linear modelling (limma), comparing lipid infusion relative to saline infusion at 300 min. Values represent log2 fold-change (FC) differences between women with polyendocrine metabolic ovarian syndrome (PMOS) and healthy controls, adjusted for within-subject saline responses. *p*-values were corrected for multiple testing using the Benjamini–Hochberg false discovery rate (FDR). Protein abbreviations: APCS, Serum amyloid P-component; ApoA1, apolipoprotein A-I; ApoE, apolipoprotein E; APP, Amyloid beta A4 protein (amyloid precursor protein); GFAP: Glial fibrillary acidic protein; IDE, insulin-degrading enzyme; MAPT, Microtubule-associated protein tau; NGF, Beta-nerve growth factor; NOG, Noggin; PAPPA, Pappalysin-1; SAA1, Serum amyloid A-1 protein; SNCA, alpha-synuclein.

#### 2.2.1. Lipid-Induced Changes in Healthy Controls

Lipid infusion resulted in a coordinated suppression of ApoA1 and all ApoE isoforms at 180 min, accompanied by reciprocal increases in insulin-degrading enzyme (IDE) and alpha synuclein (SNCA) (Figure 1; Table A3). ApoA1 (logFC −2.31; adjusted *p* = 2.09 × 10^−5^), total ApoE (−2.25; adjusted *p* = 1.25 × 10^−5^) and all ApoE isoforms (logFC −1.89 to −2.22; all adjusted *p* < 0.001) were significantly reduced following lipid exposure, while IDE (+1.23; adjusted *p* = 1.34 × 10^−5^) and SNCA (+1.08; adjusted *p* = 1.88 × 10^−4^) increased.

Between 180 and 300 min, ApoA1 (logFC +1.573, adjusted *p* = 0.0012), total ApoE (+2.29; adjusted *p* = 8.71 × 10^−6^), and all ApoE isoforms (LogFC +1.54 to +1.92; all adjusted *p* < 1.10 × 10^−5^) increased towards baseline, while IDE (−0.96; *p* = 0.0025) and SNCA (−0.75; *p* = 0.0088) declined towards pre-lipid levels (Table A4).

#### 2.2.2. Lipid-Induced Changes in PMOS

Women with PMOS exhibited the same overall temporal pattern as controls but with substantially greater effect sizes (Figure 1; Table A5). At 180 min, ApoA1 demonstrated the greatest reduction (logFC −4.31; adjusted *p* = 8.19 × 10^−15^). Total ApoE (logFC −2.92; adjusted *p* = 1.04 × 10^−9^) and all ApoE isoforms (log FC −2.17 to −2.63; all adjusted *p* < 1.04 × 10^−9^) were similarly reduced, while IDE (+1.26, adjusted *p* = 2.06 × 10^−5^) and SNCA (+0.833, adjusted *p* = 0.0019) increased.

Between 180 and 300 min, ApoA1 (logFC +2.38, adjusted *p* = 1.01 × 10^−5^), total ApoE (+1.21; adjusted *p* = 0.014) and all ApoE isoforms (logFC +0.92 to +1.13; adjusted *p*= 0.0061–0.014) increased towards baseline, while IDE (−1.03, adjusted *p* = 0.0016) and SNCA (−0.66, adjusted *p* = 0.018) declined (Table A6).

#### 2.2.3. Between-Group Comparisons and Interaction Effects

When comparing lipid-induced changes between PMOS and healthy controls at each time point (adjusted for the corresponding saline response), the strongest group difference at 180 min was for ApoAl (LogFC −2.20, adjusted *p* = 0.001), confirming more pronounced early suppression in PMOS (Table 2). Group differences for other proteins were directionally similar but did not reach FDR-adjusted significance at this early timepoint.

At 300 min, several ApoE isoforms displayed greater suppression in PMOS vs. controls (ApoE3 logFC −1.64, adjusted *p* = 0.001; ApoE4 −1.57, adjusted *p* = 0.002; ApoE −1.88, adjusted *p* = 0.003) (Table 3). These effects reflect the larger amplitude of the PMOS response rather than a difference in the underlying pattern. Baseline-to-180 min and 180-to-300-min change analyses showed patterns similar to those in the saline-adjusted comparisons (Table A7 and Table A8).

The group × time × infusion interaction terms were not statistically significant after FDR correction.

#### 2.2.4. Relationship Between ApoA1 Suppression and Metabolic Responses

**Figure A1:**
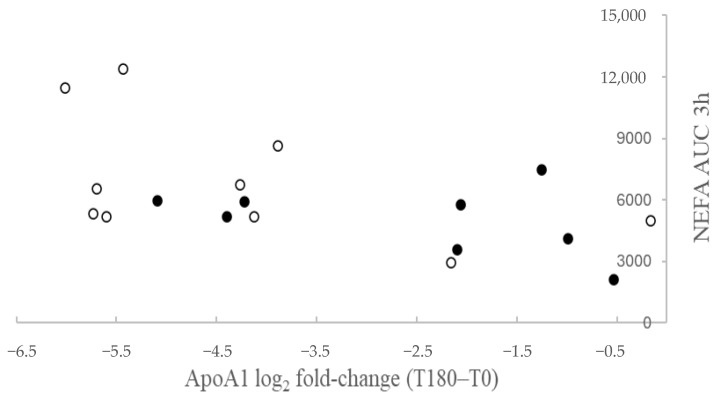
Relationship between ApoA1 log2 fold-change and NEFA AUC during lipid infusion. Greater ApoA1 suppression during lipid infusion (more negative T180-T0 values) was associated with higher NEFA AUC over the first 3 h of lipid infusion (Spearman ρ = −0.54, *p* = 0.021). Filled circles represent healthy controls and open circles represent PMOS.

The magnitude of ApoA1 suppression during lipid infusion correlated inversely with NEFA exposure over the first 3 h of lipid infusion (Spearman ρ = −0.54, *p* = 0.021). No significant associations were observed with TG AUC (ρ = 0.01, *p* = 0.98), or lipid-induced changes in glucose disposal during the clamp (ΔM; ρ ≈ 0, *p* ≈ 1.0). Scatterplot assessment showed partial separation between PMOS and healthy controls, with PMOS tending toward greater ApoA1 suppression and higher NEFA values; however, there was clear overlap and an overall inverse trend across participants (Figure A1).

## 3. Discussion

### 3.1. Summary of Key Findings

This study demonstrates that acute lipid exposure induces coordinated, transient suppression of key apolipoproteins, most notably ApoA1 and ApoE isoforms. These changes are consistent with dynamic alterations in lipid transport pathways and were observed in both healthy controls and women with PMOS. However, women with PMOS exhibited markedly amplified responses, despite preservation of the temporal pattern and reversibility, indicating that the primary distinction lies in response magnitude.

### 3.2. Characteristics of the Groups at Baseline

At baseline, women with PMOS displayed well-established metabolic differences compared with healthy controls, including higher waist-to-hip ratio, elevated HOMA-IR, and lower HDL-cholesterol [21,22]. These features are consistent with the insulin-resistant phenotype commonly described in PMOS and support the use of this cohort as a clinically relevant model of altered metabolic regulation [23,24]. Fasting triglycerides and NEFA concentrations were similar between groups, indicating that the exaggerated proteomic responses observed in PMOS cannot be attributed solely to baseline lipid levels [25]. The absence of baseline differences may be explained by the predominantly non-obese study sample and the relatively small sample size. However, the lipid challenge revealed differences in proteomic responses that were not apparent under fasting conditions.

### 3.3. Apolipoprotein Responses to Acute Lipid Stress

Acute lipid infusion induced coordinated suppression of circulating apolipoproteins, with both ApoA1 and ApoE isoforms significantly reduced at 180 min. These results are consistent with rapid remodelling of circulating lipoproteins under conditions of acute lipid excess, in which apolipoprotein distribution across lipoprotein particles may be dynamically altered [11,13].

ApoA1 is central to high-density lipoprotein (HDL) formation and reverse cholesterol transport, and its marked suppression during lipid infusion is consistent with altered lipid handling under conditions of acute lipid excess [11,13]. This reduction may be due to destabilization of HDL particles and redistribution of ApoA1 away from mature HDL fractions, a process described during acute lipid loading and consistent with altered HDL-mediated lipid trafficking [26]. Greater exposure to circulating NEFA during lipid infusion may contribute to this response, as NEFA has been shown to promote lipoprotein remodelling and alter apolipoprotein distribution, particularly under conditions of impaired suppression of lipolysis [4,11].

ApoE, which has a key role in triglyceride-rich lipoprotein metabolism and receptor-mediated lipid uptake, was similarly suppressed during lipid excess [8,11]. This may reflect coordinated downregulation of complementary lipid transport pathways during acute lipid overload [8,11]. In insulin-resistant states, the increased delivery of NEFA to the liver promotes triglyceride synthesis and very-low-density lipoprotein (VLDL) overproduction [27], which may provide a mechanistic link between lipid flux and the observed changes in ApoE-containing lipoproteins.

The coordinated suppression and differential recovery of ApoA1 and ApoE isoforms align with the established kinetics of HDL turnover and apolipoprotein exchange. ApoA1-containing HDL particles undergo rapid remodelling in response to lipid changes. In contrast, ApoE redistribution across very low-density and remnant lipoproteins has been described as part of downstream processes in lipid trafficking and clearance [8,28]. Between 180 and 300 min, both ApoA1 and ApoE isoforms increased toward baseline, indicating partial recovery during the insulin infusion. These results indicate that apolipoprotein responses to acute lipid exposure are coordinated and reversible, highlighting the importance of time-resolved assessment when interpreting proteomic responses to metabolic challenge, including acute nutritional interventions [12].

### 3.4. Integration of Apolipoprotein Responses Within Metabolic Stress

The coordinated suppression and subsequent partial recovery of ApoA1 and ApoE indicate that acute lipid exposure induces a dynamic response of the circulating lipid transport system. This pattern is consistent with the integrated adaptation of lipid transport pathways to acute metabolic stress and is in accord with coordinated remodelling of HDL-associated apolipoproteins in response to transient changes in lipid availability [11,13].

Although the overall temporal pattern was shared between groups, the magnitude of change differed. Women with PMOS exhibited substantially larger changes in apolipoprotein levels, with markedly greater suppression of relative ApoA1 abundance and overall changes approximately 2–4 times larger than in healthy controls. This suggests that metabolic stress responses are amplified rather than reflecting a fundamentally different response pattern in PMOS [1,2], consistent with impaired metabolic flexibility and altered lipid handling described in insulin-resistant states. Importantly, the absence of a significant group × time × infusion interaction indicates that the temporal sequence of apolipoprotein responses is preserved, with differences confined to response magnitude rather than pattern. In this context, identical lipid perturbations produced exaggerated changes in circulating apolipoproteins, suggesting the altered regulation of lipid handling during acute metabolic stress [15].

The experimental design allows separation of lipid-driven effects from insulin-mediated conditions. The persistence of between-group differences for ApoE isoforms at 300 min suggests that recovery towards baseline is incomplete in PMOS under hyperinsulinemic conditions. This incomplete normalization may be consistent with impaired suppression of lipolysis and sustained exposure to circulating lipids in insulin-resistant states, which may prolong disruption of lipoprotein metabolism despite insulin stimulation [29]. These findings reinforce the concept that acute elevations in circulating lipids act as a physiologically relevant metabolic stressor that elicits measurable, time-dependent proteomic responses [12,30], and suggest that dynamic metabolic challenges may reveal abnormalities in lipid handling that are not apparent under fasting conditions [12].

**Figure A2:**
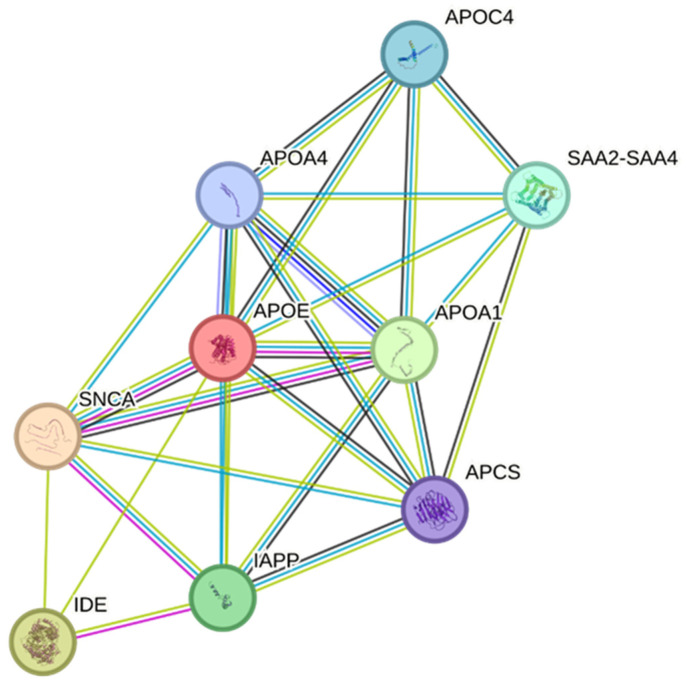
STRING protein–protein interaction network centred on lipid-responsive proteins. This network was generated by seeding STRING with proteins demonstrating significant lipid-associated responses in the present study (ApoE, ApoA1, IDE and SNCA). The figure represents interactions between the candidates and their immediate binding partners. ApoE, ApoA1, IDE and SNCA were measured in the present study. All additional nodes represent predicted interactors retrieved from STRING and were not measured in this dataset. This network is presented for descriptive purposes only, to provide biological context for the measured proteins. Different coloured edges represent different types of evidence supporting the protein-protein interactions in the STRING database.

The known biological interaction context of these lipid-responsive proteins is illustrated in Figure A2. ApoA1 and ApoE occupy central positions within interconnected lipid transport networks, supporting the interpretation that coordinated changes in these proteins reflect system-wide adaptations rather than isolated protein-specific effects [28].

### 3.5. Effects of Insulin on Lipid-Induced Proteomic Responses

The hyperinsulinemic–euglycemic clamp provided a controlled framework for assessing modulation of lipid-induced proteomic changes by insulin. Under physiological conditions, insulin suppresses lipolysis and reduces circulating lipid availability [4], thereby limiting NEFA flux and facilitating restoration of lipid homeostasis [6]. In the present study, ApoA1 and ApoE levels increased between 180 and 300 min, indicating partial reversal of lipid-induced changes during insulin stimulation. This recovery is consistent with the restoration of HDL-associated lipid transport, as inferred from changes in circulating apolipoprotein abundance and the redistribution of apolipoproteins following a reduction in circulating lipid burden [31].

ApoE isoforms remained relatively more suppressed in PMOS at 300 min, suggesting that lipid-induced changes in circulating proteins are more sustained in PMOS despite hyperinsulinemia. This pattern may be consistent with hepatic insulin resistance, in which insulin fails to adequately suppress VLDL production despite hyperinsulinemia [27]. While VLDL production was not directly measured, the persistent suppression of ApoE isoforms may be compatible with altered handling of triglyceride-rich lipoproteins under insulin-stimulated conditions. This persistence may align with impaired insulin-mediated suppression of lipid metabolism in PMOS, resulting in prolonged exposure of peripheral tissues to circulating lipids [15]. The absence of association between ApoA1 suppression and lipid-induced changes in glucose disposal (ΔM) suggests that these proteomic responses may reflect aspects of lipid handling that are not fully captured by clamp-derived measures of insulin sensitivity. These findings suggest that dynamic proteomic responses may reflect aspects of metabolic regulation that are not fully captured by conventional measures of insulin sensitivity [12,32].

Overall, these findings suggest that insulin-mediated recovery of apolipoproteins may provide additional insight into metabolic resilience beyond conventional measures of insulin sensitivity.

### 3.6. Translational Implications

The current findings suggest that dynamic proteomic responses to acute lipid challenge may have translational relevance following further validation. Current clinical assessment of metabolic risk relies predominantly on fasting biomarkers, which do not capture dynamic impairments in lipid handling [33,34,35].

The magnitude of ApoA1 suppression and the coordinated responses of the ApoE-ApoA1 axis may represent candidate indicators of lipid handling and acute lipid stress responses [33,34,35,36,37,38]. However, further studies will be required to determine whether these dynamic protein responses have utility as biomarkers of metabolic regulation and response to metabolic stress [39].

### 3.7. Strengths and Limitations

This study has several strengths. The integration of high-throughput proteomics with controlled lipid infusion and hyperinsulinemic–euglycemic clamp enabled investigation of dynamic metabolic responses in vivo [6,12,40]. The randomized cross-over design, with each participant as their own control, minimized inter-individual variability and strengthened internal validity [12]. This approach allowed the separation of lipid-driven effects from insulin-mediated reversibility under stable glycemic conditions. Repeated sampling across defined time points enabled assessment of temporal dynamics, supporting mechanistic interpretation of coordinated apolipoprotein responses [12]. The inclusion of a saline control and fasting conditions reduced confounding and supports the interpretation that the observed proteomic changes were lipid-specific.

Several limitations should be acknowledged. The sample size was small, although consistent with metabolic clamp studies [6], and no direct measures of lipoprotein particle kinetics or remnant flux were obtained [41,42]. In addition, HDL functionality and hepatic lipoprotein production were not assessed directly. Consequently, the mechanistic interpretations presented are based on biologically plausible explanations rather than direct observations from the present study. Circulating apolipoprotein concentrations reflect the net effects of secretion, redistribution, and clearance. The proteomic platform provides measures of relative abundance rather than absolute concentration or functional activity [43], and isoform-specific findings require biochemical validation. Consequently, the observed log_2_ fold-changes should be interpreted as relative changes in SOMAscan signal intensity rather than absolute changes in circulating protein concentration. The study population comprised relatively young, premenopausal women with a severe, well-defined PMOS phenotype, which may limit generalizability to older or postmenopausal women and milder metabolic phenotypes (B, C, D) [17]. Correlation analyses were exploratory and limited by the small sample size; consequently, the observed association between ApoA1 suppression and NEFA should be interpreted cautiously until confirmed in larger independent studies.

The findings should be interpreted as exploratory. The observed apolipoprotein responses provide insight into the dynamic regulation of lipid metabolism under controlled conditions, but their clinical utility as biomarkers of metabolic function requires further validation. Larger studies incorporating repeated measurements in free-living settings and across broader populations will be required to confirm the magnitude, reproducibility and generalizability of these responses, as well as their relevance for assessing metabolic health and response to intervention.

## 4. Materials and Methods

### 4.1. Design

This exploratory study was designed around an optimal experimental paradigm: randomized crossover lipid versus saline infusion followed by a hyperinsulinemic–euglycemic clamp, with repeated blood sampling. The study protocol was approved by the Leeds (Central) Research Ethics Committee (reference 10/H1313/44). The study was conducted in accordance with the principles of the Declaration of Helsinki. Written informed consent was obtained from all participants before their involvement.

Each participant underwent two infusion studies (saline and lipid), each followed by a hyperinsulinemic–euglycemic clamp. The infusions were spaced 7 days apart to minimize carry-over effects.

This study was conducted in the same participants as our previously published cardiovascular-risk proteins article [20]. Here we focus on a targeted subset of lipid- and insulin-responsive plasma proteins derived from a distinct SOMAscan panel and analyzed using a predefined system-level framework.

### 4.2. Participants

Twelve women with PMOS were recruited from local endocrine clinics, and 10 healthy control women were recruited through advertisements at Hull University and East Yorkshire Hospital Trust newsletters. A formal sample size calculation was not performed. The sample size was based on feasibility and was comparable to previous clamp-based studies that used intensive physiological protocols and repeated blood sampling [6]. The study was designed to investigate protein responses to acute metabolic challenge rather than to estimate population prevalence. PMOS was diagnosed by the Rotterdam criteria (≥2 of oligomenorrhoea, hyperandrogenism, or polycystic ovaries on ultrasound) [17]. Inclusion and exclusion criteria have been reported in detail previously [20].

### 4.3. Protocol

**Figure 2:**
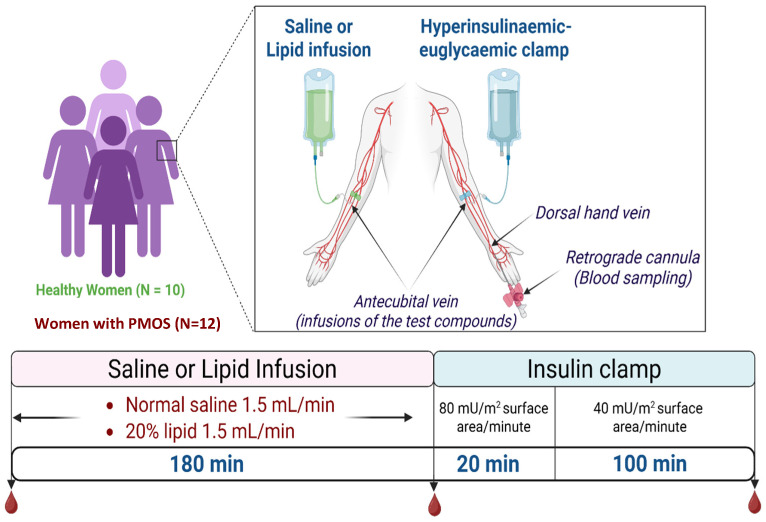
Study protocol and timing of metabolic interventions. Healthy control women and women with polyendocrine metabolic ovarian syndrome (PMOS) underwent a randomized cross-over protocol consisting of a 5-h saline or lipid infusion. A hyperinsulinemic–euglycemic clamp was initiated at 180 min and continued for 120 min to assess insulin-mediated reversibility of lipid-induced proteomic changes. Blood samples for plasma proteomics were collected at baseline and at 180 and 300 min.

The study protocol has been published previously [20] and is illustrated in Figure 2. Briefly, participants attended after an overnight fast. Saline (0.9% NaCl) or 20% lipid emulsion (soybean oil, egg phospholipids, glycerol; Fresenius Kabi; Cheshire, UK) was infused at 1.5 mL/min for 5 h, with unfractionated heparin (0.3 U/kg/min) co-infused during lipid infusion. Three hours after infusion onset, a 2-h hyperinsulinemic–euglycemic clamp was performed using intravenous insulin (Humulin S; Eli Lilly, Basingstoke, United Kingdom) at 80 mU/m^2^/min (20 min) followed by 40 mU/m^2^/min. Plasma glucose was maintained at 5.0 mmol/L by variable 20% dextrose infusion, adjusted at 5 min intervals. The glucose disposal rate (M-value) was calculated as described by DeFronzo et al. [6].

### 4.4. Blood Sample Collection and Biochemical Analysis

Blood was collected at baseline (T0), 180 min (T180), and 300 min (T300). Samples were centrifuged and stored at −80 °C. Biochemical assays for glucose, insulin, triglycerides, and NEFA were performed as described previously [20].

### 4.5. SomaScan Proteomics

The SOMAscan proteomics assay [43] was used to measure a targeted panel of 15 plasma proteins (Appendix A: Table A1). The present study focused on a pre-specified subset of proteins selected a priori from the larger SOMAscan dataset based on their established roles in lipid biology, lipoprotein metabolism, associated inflammatory processes, and responsiveness to lipid exposure and insulin-sensitive pathways [11]. The methodology was performed as previously described by Moin et al. [44].

### 4.6. Systems Biology Analysis

STRING (Search Tool for the Retrieval of Interacting Genes, version 12.0; https://string-db.org/) was utilized to generate protein–protein interaction networks (PPIN).

### 4.7. Data Analysis and Statistics

The Homeostatic Model Assessment of Insulin Resistance (HOMA-IR) was calculated as described previously [45]. Area under the curve (AUC) was determined for NEFA, insulin, glucose and triglycerides (TG) [46].

As this was an exploratory physiological study, a formal sample size calculation was not performed; however, the number of patients is in accord with previous studies [6,47]. Data distributions were assessed for normality using GraphPad Prism (Version 10.4.0, GraphPad Software, San Diego, CA, USA). Normally distributed variables are presented as mean ± SD, whereas skewed variables are presented as median (interquartile range).

Between-group comparisons of baseline clinical and biochemical variables were performed using independent *t*-tests for normally distributed variables or Mann–Whitney U tests for skewed variables. Where within-subject comparisons were performed, paired *t*-tests were applied. A *p* value ≤ 0.05 was considered statistically significant. Statistical analyses were performed using GraphPad Prism (Version 10.4.0, San Diego, CA, USA).

For SomaScan proteomics, statistical analyses were performed on log2 RFU values using R version 3.5.2 (R Foundation for Statistical Computing, Vienna, Austria). Differential protein abundance was analyzed using the autonomics pipeline and the limma package (v3.64.3) in R (v4.4.1) [48]. Limma-obtained *p*-values were corrected using the Benjamini–Hochberg method [49]. Each participant was treated as a blocking factor to account for repeated measures. Protein intensities were log2-transformed and normalized before modelling. Primary contrasts were defined to assess between-treatment and between-group differences at 180 min and 300 min. Further difference-in-differences strategies were examined using interaction terms (Group × Infusion × Time). Specifically, changes during lipid infusion (0–180 min) were compared with the corresponding changes during saline (0–180 min), and changes during insulin infusion following lipid (0–300 min) were compared with those during saline (0–300 min). This approach controlled for any temporal drift or non-specific procedural effects during saline infusion. Statistical significance for proteomic analyses was defined using Benjamini–Hochberg FDR-adjusted *p* values < 0.05. Fold changes are reported as log_2_ fold-change (logFC) to indicate effect size. Exploratory Spearman correlation analyses were performed between participant-level ApoA1 log_2_ fold-change during lipid infusion (0–180 min) and NEFA AUC, TG AUC and the lipid–saline difference in glucose disposal ΔM. Complete data were available for all participants at all study time points, and no imputation for missing data was required.

## 5. Conclusions

Controlled lipid exposure induces coordinated and reversible suppression of HDL-associated apolipoproteins, including ApoA1 and ApoE isoforms. This response is preserved in women with PMOS but occurs with greater magnitude, suggesting amplification of the metabolic stress response.

Despite partial recovery during hyperinsulinemia, incomplete normalization in PMOS suggests more sustained disruption of circulating apolipoprotein responses under insulin-stimulated conditions. These findings suggest that dynamic proteomic responses to lipid stress may provide additional insight into metabolic responses that are not apparent from static fasting measures.

## Figures and Tables

**Table 1:** Baseline characteristics of healthy control women and women with polyendocrine metabolic ovarian syndrome.

Parameters	Controls (*n* = 10)	PMOS (*n* = 12)	*p*-Value
Age (years)	25.3 ± 6.5	28.3 ± 6.5	0.290
BMI (kg/m^2^)	26.8 ± 6.5	29.4 ± 5.5	0.320
Waist (cm)	81.1 ± 14.2	98.8 ± 15.8	0.013
WHR	0.79 ± 0.06	0.87 ± 0.06	0.006
FPG (mmol/L)	4.89 ± 0.56	4.94 ± 0.55	0.840
HbA1c (mmol/mol)	33 ± 5.6	34 ± 2.9	0.596
HOMA-IR	1.34 (0.80, 2.13)	2.30 (1.30, 3.90)	0.041
TC (mmol/L)	4.61 ± 0.75	4.13 ± 0.65	0.123
TG (mmol/L)	0.84 ± 0.18	1.25 ± 0.72	0.096
HDL-C (mmol/L)	1.48 ± 0.47	1.12 ± 0.20	0.026
LDL-C (mmol/L)	2.66 ± 0.56	2.33 ± 0.51	0.164

Values are presented as mean ± SD for normally distributed variables and median (interquartile range) for skewed variables. Between-group comparisons were performed using independent *t*-tests for normally distributed variables, Mann–Whitney U tests for skewed variables and χ^2^ tests for categorical variables. All measurements were obtained under fasting baseline conditions prior to infusion protocols. Abbreviations: BMI, body mass index; WHR, waist-to-hip ratio; FPG, fasting plasma glucose; HbA1c, glycated hemoglobin; HOMA-IR, homeostatic model assessment of insulin resistance; TC, total cholesterol; TG, triglycerides; HDL-C, high-density lipoprotein cholesterol; LDL-C, low-density lipoprotein cholesterol; PMOS, polyendocrine metabolic ovarian syndrome.

## Data Availability

The original contributions presented in this study are included in the article. Further inquiries can be directed to the corresponding author.

## Abbreviations

The following abbreviations are used in this manuscript:

ApoA1	Apolipoprotein A-1
ApoE	Apolipoprotein E
ApoE2/ApoE3/ApoE4	Apoplipoprotein E isoforms
AUC	Area under the curve
BMI	Body mass index
FDR	False discovery rate
FPG	Fasting blood glucose
HDL	High-density lipoprotein
HDL-C	High-density lipoprotein cholesterol
HOMA-IR	Homeostatic Model of Assessment of Insulin Resistance
IDE	Insulin-degrading enzyme
logFC	Log_2_ fold-change
M-value (M)	Glucose disposal rate during hyperinsulinemic–euglycemic clamp
NEFA	Non-esterified fatty acids
PMOS	Polyendocrine metabolic ovarian syndrome
PPIN	Protein–protein interaction network
SNCA	Alpha-synuclein
SOMAscan	Aptamer-based proteomics assay platform
STRING	Search Tool for the Retrieval of Interacting Genes
TG	Triglycerides
VLDL	Very low-density lipoprotein
WHR	Waist-to-hip ratio

## Appendix A

**Table A1:** Targeted lipid- and insulin-responsive plasma proteins analyzed using the SOMAscan platform.

Protein	Target Full Name
APP	Amyloid beta A4 protein (amyloid precursor protein)
SNCA	Alpha-synuclein
APCS	Serum amyloid P-component
PAPPA	Pappalysin-1
MAPT	Microtubule-associated protein tau
ApoE	Apolipoprotein E
ApoE2	Apolipoprotein E (isoform E2)
ApoE3	Apolipoprotein E (isoform E3)
ApoE4	Apolipoprotein E (isoform E4)
SAA1	Serum amyloid A-1 protein
NOG	Noggin
ApoA1	Apolipoprotein A-I
IDE	Insulin-degrading enzyme
NGF	Beta-nerve growth factor
GFAP	Glial fibrillary acidic protein

Proteins are listed using protein nomenclature to distinguish measured analytes from gene symbols.

## Appendix B

**Table A2:** Effect of lipid infusion on insulin sensitivity assessed during hyperinsulinemic–euglycemic clamp.

	Controls (*n* = 10)			PMOS (*n* = 12)		
Parameters	Saline	Lipid	*p*-Value	Saline	Lipid	*p*-Value
Glucose disposal (mg/kg/min)	5.25 (3.30, 6.48)	2.6 (0.88, 3.9)	<0.001	3.15 (2.94, 3.9)	1.06 (0.72, 1.43)	<0.001
TG AUC 3 h (mmol/L)	2.34 ± 0.14	11.0 ± 1.28	<0.001	3.75 ± 9.64	12.98 ± 1.65	0.01
NEFA AUC 3 h (mmol/L)	1.81 (1.43, 2.2)	6.23 (5.1, 7.7)	0.005	1.88 (1.50, 2.06)	6.66 (4.67, 8.46)	0.01
Insulin AUC 3 h (pmol/L)	85 (60, 190)	94 (50, 138)	0.51	161 (111, 250)	170 (69, 344)	–
TG AUC 5 h (mmol/L)	3.78 ± 0.86	22.5 ± 7.8	<0.001	5.29 ± 2.99	23.9 ± 11.4	<0.001
NEFA AUC 5 h (mmol/L)	2.24 (1.83, 2.7)	12.04 (8.56, 13.5)	0.005	2.26 (1.8, 2.55)	12.4 (9.25, 1.44)	<0.001

Skewed variables are presented as median (25th, 75th percentile), and normally distributed variables as mean ± SEM. Insulin sensitivity is expressed as glucose infusion rate derived from the hyperinsulinemic–euglycemic clamp. Triglycerides (TG) and non-esterified fatty acids (NEFA) are summarized as area under the curve (AUC), calculated using the trapezoidal method. PMOS, polyendocrine metabolic ovarian syndrome. Significant comparisons (*p* ≤ 0.05) are reported with exact *p* values; ‘–’ indicates non-significant comparisons (*p* > 0.05). These data have been published previously and are included here for physiological context [5].

**Table A3:** Baseline-to-180-min changes in protein levels during lipid infusion in healthy control women.

Target	logFC	AveExpr	t	*p*. Value	adj. *p*. Value	B
ApoE4	−2.21813	16.81688	−6.01302	2.65 × 10^−8^	3.63 × 10^−7^	8.683574
ApoE2	−1.89269	17.31291	−5.88135	4.84 × 10^−8^	3.63 × 10^−7^	8.101432
ApoE3	−2.05296	16.68361	−5.57473	1.92 × 10^−7^	9.59 × 10^−7^	6.771376
ApoE	−2.24759	13.90599	−4.91449	3.27 × 10^−6^	1.23 × 10^−5^	4.044187
ApoA1	−2.31272	12.74753	−4.73039	6.97 × 10^−6^	2.09 × 10^−5^	3.321125
IDE	1.230609	10.99177	4.209666	5.39 × 10^−5^	0.000134772	1.375917
SNCA	1.079344	13.17015	4.079376	8.79 × 10^−5^	0.000188297	0.913999
SAA1	−1.12824	9.392361	−1.25478	0.212331	0.390712111	−6.00064
MAPT	−0.29695	6.908749	−1.19587	0.234427	0.390712111	−6.07156
APP	0.412797	15.05522	1.051662	0.295356	0.443034209	−6.23114
NGF	−0.43603	9.18805	−0.90391	0.368101	0.501955231	−6.37381
GFAP	−0.0981	8.658668	−0.62095	0.535973	0.639014533	−6.5876
APCS	−0.11875	14.94684	−0.59397	0.553813	0.639014533	−6.60386
NOG	0.115944	11.74102	0.27186	0.786261	0.803751746	−6.74258
PAPPA	−0.06342	13.2841	−0.24912	0.803752	0.803751746	−6.74848

Changes in protein levels are expressed as log2 fold-change. Statistical comparisons were performed using linear modelling, with significance assessed at *p* ≤ 0.05. *p*-values were adjusted for multiple testing using the Benjamini–Hochberg false discovery rate (FDR). B, log-odds that a protein is differentially expressed, were estimated using empirical Bayes moderation (limma). Protein abbreviations: APCS, Serum amyloid P-component; ApoA1, apolipoprotein A-I; ApoE, apolipoprotein E; APP, Amyloid beta A4 protein (amyloid precursor protein); GFAP: Glial fibrillary acidic protein; IDE, insulin-degrading enzyme; MAPT, Microtubule-associated protein tau; NGF, Beta-nerve growth factor; NOG, Noggin; PAPPA, Pappalysin-1; SAA1, Serum amyloid A-1 protein; SNCA, α-synuclein.

**Table A4:** 180-to-300-min changes in protein levels during lipid infusion in healthy control women.

Target	logFC	AveExpr	t	*p*. Value	adj. *p*. Value	B
ApoE4	1.923469	16.81688	5.374706	4.62 × 10^−7^	6.93 × 10^−6^	5.99033
ApoE	2.289758	13.90599	5.160778	1.16 × 10^−6^	8.71 × 10^−6^	5.107541
ApoE3	1.770839	16.68361	4.956622	2.75 × 10^−6^	1.10 × 10^−5^	4.284925
ApoE2	1.542441	17.31291	4.940496	2.94 × 10^−6^	1.10 × 10^−5^	4.220811
IDE	−0.9751	10.99177	−3.43827	0.000839	0.002517	−1.10593
ApoA1	1.573748	12.74753	3.317973	0.001245	0.003112	−1.46963
SNCA	−0.75337	13.17015	−2.93501	0.004093	0.008771	−2.55642
PAPPA	0.291205	13.2841	1.179103	0.241008	0.45189	−5.98114
MAPT	0.251473	6.908749	1.043899	0.298916	0.498194	−6.12867
SAA1	0.848268	9.392361	0.972442	0.333054	0.49958	−6.19955
NGF	0.149739	9.18805	0.319967	0.749626	0.913489	−6.61633
GFAP	0.048178	8.658668	0.314358	0.75387	0.913489	−6.61809
NOG	0.109555	11.74102	0.264786	0.791691	0.913489	−6.63234
APP	0.067419	15.05522	0.177046	0.859812	0.921227	−6.65159
APCS	−0.00797	14.94684	−0.04109	0.967301	0.967301	−6.66632

Changes in protein levels are expressed as log2 fold-change. Statistical comparisons were performed using linear modelling, with significance assessed at *p* ≤ 0.05. *p*-values were adjusted for multiple testing using the Benjamini–Hochberg false discovery rate (FDR). B, log-odds that a protein is differentially expressed, were estimated using empirical Bayes moderation (limma). Protein abbreviations: APCS, Serum amyloid P-component; ApoA1, apolipoprotein A-I; ApoE, apolipoprotein E; APP, Amyloid beta A4 protein (amyloid precursor protein); GFAP: Glial fibrillary acidic protein; IDE, insulin-degrading enzyme; MAPT, Microtubule-associated protein tau; NGF, Beta-nerve growth factor; NOG, Noggin; PAPPA, Pappalysin-1; SAA1, Serum amyloid A-1 protein; SNCA, α-synuclein.

**Table A5:** Baseline-to-180-min changes in protein levels during lipid infusion in women with polyendocrine metabolic ovarian syndrome.

Target	logFC	AveExpr	t	*p*. Value	adj. *p*. Value	B
ApoA1	−4.30527	12.74753	−9.5679	5.46 × 10^−16^	8.19 × 10^−15^	25.79697
ApoE4	−2.63384	16.81688	−7.75778	5.76 × 10^−12^	4.32 × 10^−11^	16.64243
ApoE2	−2.1679	17.31291	−7.31949	5.12 × 10^−11^	2.56 × 10^−10^	14.49122
ApoE3	−2.35642	16.68361	−6.95247	3.09 × 10^−10^	1.04 × 10^−9^	12.72111
ApoE	−2.91653	13.90599	−6.929	3.47 × 10^−10^	1.04 × 10^−9^	12.60904
IDE	1.261602	10.99177	4.689138	8.24 × 10^−6^	2.06 × 10^−5^	2.781956
SNCA	0.833668	13.17015	3.423498	0.000881	0.001888	−1.66374
MAPT	−0.4521	6.908749	−1.97825	0.050506	0.094699	−5.31422
APP	0.663195	15.05522	1.835798	0.069202	0.108255	−5.57794
SAA1	−1.50302	9.392361	−1.81625	0.07217	0.108255	−5.61269
APCS	−0.23381	14.94684	−1.27068	0.206663	0.281813	−6.43916
NGF	−0.40736	9.18805	−0.91755	0.360948	0.430955	−6.82276
PAPPA	−0.2094	13.2841	−0.89374	0.373494	0.430955	−6.84425
NOG	0.130117	11.74102	0.331495	0.740928	0.793851	−7.18872
GFAP	−0.03718	8.658668	−0.25571	0.798675	0.798675	−7.21105

Changes in protein levels are expressed as log2 fold-change. Statistical comparisons were performed using linear modelling, with significance assessed at *p* ≤ 0.05. *p*-values were adjusted for multiple testing using the Benjamini–Hochberg false discovery rate (FDR). B, log-odds that a protein is differentially expressed, were estimated using empirical Bayes moderation (limma). Protein abbreviations: APCS, Serum amyloid P-component; ApoA1, apolipoprotein A-I; ApoE, apolipoprotein E; APP, Amyloid beta A4 protein (amyloid precursor protein); GFAP: Glial fibrillary acidic protein; IDE, insulin-degrading enzyme; MAPT, Microtubule-associated protein tau; NGF, Beta-nerve growth factor; NOG, Noggin; PAPPA, Pappalysin-1; SAA1, Serum amyloid A-1 protein; SNCA, α-synuclein.

**Table A6:** 180-to-300-min changes in protein levels during lipid infusion in women with polyendocrine metabolic ovarian syndrome.

Samples	logFC	AveExpr	t	*p*. Value	adj. *p*. Value	B
ApoA1	2.379195	12.74753	5.287452	6.74 × 10^−7^	1.01 × 10^−5^	5.636741
IDE	−1.03146	10.99177	−3.83373	0.000215	0.001612	0.173178
ApoE4	1.129161	16.81688	3.325861	0.001213	0.006066	−1.43267
ApoE2	0.920438	17.31291	3.107674	0.002423	0.009087	−2.06628
ApoE	1.21437	13.90599	2.885063	0.004745	0.014062	−2.67577
ApoE3	0.958101	16.68361	2.82681	0.005625	0.014062	−2.82891
SNCA	−0.65649	13.17015	−2.69592	0.008171	0.017508	−3.16321
PAPPA	0.276886	13.2841	1.181767	0.239954	0.449913	−5.96205
MAPT	0.220475	6.908749	0.964729	0.336885	0.561476	−6.19078
APP	−0.26823	15.05522	−0.7425	0.459434	0.689151	−6.3778
SAA1	0.549263	9.392361	0.663728	0.508312	0.692918	−6.43254
GFAP	−0.08624	8.658668	−0.59317	0.554334	0.692918	−6.4764
NGF	0.19664	9.18805	0.442914	0.658734	0.760078	−6.55348
APCS	0.04565	14.94684	0.248093	0.804549	0.862017	−6.62023
NOG	−0.03885	11.74102	−0.09899	0.921337	0.921337	−6.64592

Changes in protein levels are expressed as log2 fold-change. Statistical comparisons were performed using linear modelling, with significance assessed at *p* ≤ 0.05. *p*-values were adjusted for multiple testing using the Benjamini–Hochberg false discovery rate (FDR). B, log-odds that a protein is differentially expressed, were estimated using empirical Bayes moderation (limma). Protein abbreviations: APCS, Serum amyloid P-component; ApoA1, apolipoprotein A-I; ApoE, apolipoprotein E; APP, Amyloid beta A4 protein (amyloid precursor protein); GFAP: Glial fibrillary acidic protein; IDE, insulin-degrading enzyme; MAPT, Microtubule-associated protein tau; NGF, Beta-nerve growth factor; NOG, Noggin; PAPPA, Pappalysin-1; SAA1, Serum amyloid A-1 protein; SNCA, α-synuclein.

**Table A7:** Differential baseline-to-180-min response of targeted plasma proteins to lipid infusion in women with PMOS compared with healthy controls.

Samples	logFC	AveExpr	t	Adj. *p*-Value (FDR)
ApoA1	−2.06	12.75	−2.22	0.03
ApoE	−0.93	13.91	−1.07	0.28
APP	0.65	15.06	0.87	0.39
ApoE4	−0.59	16.82	−0.84	0.40
ApoE3	−0.54	16.68	−0.77	0.44
ApoE2	−0.41	17.31	−0.67	0.50
SNCA	−0.30	13.17	−0.60	0.55
PAPPA	−0.22	13.28	−0.45	0.66
GFAP	0.07	8.66	0.24	0.81
IDE	0.11	10.99	0.20	0.84
SAA1	−0.10	9.39	−0.06	0.95
APCS	0.02	14.95	0.05	0.96
MAPT	−0.02	6.91	−0.04	0.96
NGF	−0.03	9.19	−0.03	0.97
NOG	0.02	11.74	0.02	0.98

Differential protein responses represent baseline-to-180-min changes during lipid infusion, estimated using linear modelling (limma). Values reflect log2 fold-change (FC) differences between women with polyendocrine metabolic ovarian syndrome (PMOS) and healthy controls. *p*-values were corrected for multiple testing using the Benjamini–Hochberg false discovery rate (FDR). PMOS, polyendocrine metabolic ovarian syndrome. Protein abbreviations: APCS, Serum amyloid P-component; ApoA1, apolipoprotein A-I; ApoE, apolipoprotein E; APP, Amyloid beta A4 protein (amyloid precursor protein); GFAP: Glial fibrillary acidic protein; IDE, insulin-degrading enzyme; MAPT, Microtubule-associated protein tau; NGF, Beta-nerve growth factor; NOG, Noggin; PAPPA, Pappalysin-1; SAA1, Serum amyloid A-1 protein; SNCA, alpha-synuclein.

**Table A8:** Differential 180-to-300-min response of targeted plasma proteins to lipid infusion in women with PMOS compared with healthy controls.

Samples	logFC	AveExpr	t	Adj. *p*-Value (FDR)
ApoE	−1.97	13.91	−2.27	0.03
ApoE3	−1.49	16.68	−2.13	0.04
ApoE4	−1.43	16.82	−2.04	0.04
ApoE2	−1.09	17.31	−1.79	0.08
ApoA1	−1.12	12.75	−1.20	0.23
APP	0.77	15.06	1.03	0.31
SNCA	−0.36	13.17	−0.72	0.48
PAPPA	−0.18	13.28	−0.37	0.71
APCS	0.14	14.95	0.35	0.72
NOG	0.15	11.74	0.19	0.85
SAA1	−0.30	9.39	−0.17	0.86
MAPT	0.08	6.91	0.17	0.87
NGF	0.13	9.19	0.15	0.88
IDE	−0.05	10.99	−0.10	0.92
GFAP	−0.03	8.66	−0.09	0.93

Differential protein responses represent 180-to-300-min changes during lipid infusion, estimated using linear modelling (limma). Values reflect log2 fold-change (FC) differences between women with polyendocrine metabolic ovarian syndrome (PMOS) and healthy controls. *p*-values were corrected for multiple testing using the Benjamini–Hochberg false discovery rate (FDR). PMOS, polyendocrine metabolic ovarian syndrome. Protein abbreviations: APCS, Serum amyloid P-component; ApoA1, apolipoprotein A-I; ApoE, apolipoprotein E; APP, Amyloid beta A4 protein (amyloid precursor protein); GFAP: Glial fibrillary acidic protein; IDE, insulin-degrading enzyme; MAPT, Microtubule-associated protein tau; NGF, Beta-nerve growth factor; NOG, Noggin; PAPPA, Pappalysin-1; SAA1, Serum amyloid A-1 protein; SNCA, alpha-synuclein.
